# Low-Dose IL-2 Attenuated Depression-like Behaviors and Pathological Changes through Restoring the Balances between IL-6 and TGF-β and between Th17 and Treg in a Chronic Stress-Induced Mouse Model of Depression

**DOI:** 10.3390/ijms232213856

**Published:** 2022-11-10

**Authors:** Chengyi Huang, Fucheng Zhang, Peng Li, Cai Song

**Affiliations:** 1 Research Institute for Marine Drugs and Nutrition, College of Food Science and Technology, Guangdong Ocean University, Zhanjiang 524088, China; 2 Marine Medicine and Development Center, Shenzhen Institute of Guangdong Ocean University, Shenzhen 518120, China; 3 Guangdong Provincial Key Laboratory of Aquatic Product Processing and Safety, College of Food Science and Technology, Guangdong Ocean University, Zhanjiang 524088, China; 4 Affiliated Hospital of Guangdong Medical University, Zhanjiang 524088, China

**Keywords:** IL-2, Th17, Treg, IL-6, TGF-β, CUMS, depression

## Abstract

Microglia activation, increased IL-6 and decreased TGF-β were found in depressed patients or in animal models of depression. IL-6 enhances T helper 17 cell differentiation, thereby causing an imbalance between Th17 and Treg cells, which induces neuroinflammation and neuronal dysfunction. However, whether imbalances between IL-6 and TGF-β and between Th17 and Treg occur in depression and whether depression can be improved upon restoring these imbalances are unknown. Treg promoter IL-2 (1500UI/0.1 mL/day) was used to treat a mouse model of depression induced by chronic unpredictable mild stress (CUMS). The behavior and concentrations of IL-6, TGF-β, Th17, IL-17A, IL-17Rc, Treg-related factors (helios and STAT5), astrocyte A1 phenotype S100β, microglia M1 phenotype Iba-1, indoleamine-2,3-dioxygenase (IDO) enzyme, corticosterone (CORT) and neurotransmitters were evaluated. When compared to controls, CUMS reduced sucrose preference, the number of entries into and the time spent in the open arms of the elevated plus maze and the exploration in the “open field”, while it increased the immobility time in tail suspension, which was ameliorated by IL-2 treatment. RoRα, S100β, IL-17A, IL-17Rc, IL-6, Iba-1, IDO enzyme and CORT concentrations were significantly increased, and Helios, FoxP3^+^, STAT5 and TGF-β were significantly decreased by CUMS, which were significantly attenuated by IL-2 when compared to the CUMS group. The NE, DA and 5-HT contents and those of their metabolites were decreased by CUMS, which returned to control levels after IL-2 treatment. The study demonstrated that imbalances between IL-6 and TGF-β and between Th17and Treg occurred in the hippocampus of the depression model. IL-2 attenuated depression- and anxiety-like behaviors and normalized the neurotransmitter concentration and the activity of the IDO enzyme, astrocytes and microglia through restoring both balances, but it did not decrease the CORT concentration.

## 1. Introduction

The hypotheses of depression, including hypothalamic-pituitary-adrenal (HPA) axis dysfunction [1] and monoamine neurotransmitter deficiency [2], have been extensively studied. Since 1995, the macrophage/T lymphocyte hypothesis of depression has been gradually demonstrated. Clinical investigations reported the activation of macrophages and lymphocyte subtypes, such as M1, M2, T help (h)1 and Th17 cells, and their excessively secreted pro-inflammatory cytokines, such as interleukin (IL)-1, IL-6, interferon (IFN)-γ and tumor necrosis factor (TNF)-α, in patients with major depression [3]. Our team has reported that the chronic administration of proinflammatory cytokines, such as IL-1β or IL-6, can induce: (1) corticosterone (CORT) secretion and HPA axis dysfunction [4]; (2) the activation of the inflammatory phenotype of microglia [5] and astrocytes [6]; (3) an increase in oxidants and an inhibition of neurotropic factor production, which causes neuronal apoptosis; (4) the activation of indoleamine-2,3-dioxygenase (IDO) in the periphery and brain, which reduces the synthesis of norepinephrine (NE) and 5-hydroxytryptamine (5-HT) [7]. All of these can contribute to depression.

The innate immune inflammatory response is dominated by macrophages, which further activate acquired immunity, such as pro- and anti-inflammatory T lymphocyte subtypes. In a normal immune system, M1 and M2 macrophage phenotypes release proinflammatory and anti-inflammatory cytokines, such as IL-6 and TGF-β, respectively, which induce the differentiation of Th17 and Treg [8,9]. Moreover, activated microglia can produce T cells-migrating chemokines, which promote Th17 cell differentiation and migration to the brain [10]. In turn, Th17-released IL-17A binds to the specific receptor IL-17 receptor c (R) on the surface of microglia and astrocytes [11], which further activates the microglia M1 phenotype, astrocyte A1 phenotype and neuroinflammation [12]. Céline Meyer et al. reported that the existence of lymphatic vessels in the brain is closely related to the infiltration of T lymphocytes into the brain, triggering neuroinflammation [13].

In contrast to the activation of Th1 and Th17 lymphocytes, we and others reported that lymphocyte proliferation, lymphocyte growth factor and Treg differential factor IL-2 were decreased in depressed patients [14,15,16]. Low-dose IL-2 administration was found to attenuate inflammatory responses and anxiety-like behaviors [17,18,19] in patients with autoimmune diseases, as well as attenuate depression-like behaviors in a depression model of olfactory bulbectomized rats [20]. However, there are two questions: (1) whether the imbalances between IL-6 and TGF-β and between Th17 and Treg occur in depression; and (2) whether low-dose IL-2 can improve depression by restoring these imbalances. To answer these two questions, the present study hypothesized that chronic stress stimulates the HPA axis and increases the CORT level, which over-activates microglia and astrocytes and destroys the balance between IL-6 and TGF-β and between Th17 and Treg in the brain. Excessive IL-17A combines with the specific receptor IL-17Rc in microglia and astrocytes, further activates microglia M1 phenotype and astrocyte A1 phenotype and triggers IDO enzyme activity, thereby causing neurotransmitter metabolism and inducing depression-like behaviors. Low-dose IL-2 can reverse these changes by balancing two pairs of lymphocyte subtypes.

**Figure 1:**
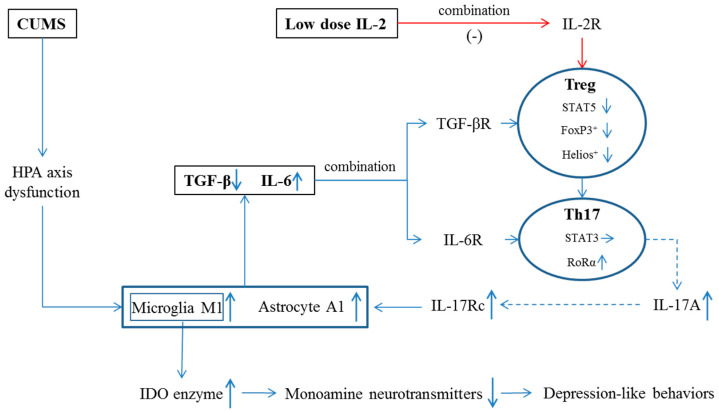
Possible mechanism by which low-dose IL-2 reversed CUMS-induced depression-like changes. IL-2R, interleukin-2 receptor; TGF-βR, transforming growth factor-β receptor; IL-6R, IL-6 receptor, IL-17Rc, IL-17 receptor c; ↑, level was increased; ↓, level was decreased; →, level was unchanged; solid line, known evidence; dotted line, hypothesis.

In order to demonstrate these hypotheses, IL-2 was selected to treat a popular rodent model of depression induced by chronic unpredictable mild stress (CUMS), simulating long-term pressures in human beings. Our previous studies have demonstrated that CUMS-induced changes in behaviors, the HPA axis, neurotransmission and immune functions are similar to those observed in patients with major depression. Animal anxiety- and depression-like behaviors were measured by sucrose preference, “open field” [21], elevated plus maze [22] and tail suspension tests [23]. Then, the concentration of monoamine neurotransmitters and their metabolites in the hippocampus and amygdala were measured by high-performance liquid chromatography (HPLC). IL-6 and TGF-β were assayed in the hippocampus by quantitative real-time polymerase chain reaction (q-PCR) and an enzyme-linked immunosorbent assay (ELISA). IDO enzyme and CORT were also detected by ELISA. To explore the relationship between glia cells (microglia and astrocytes) activation and lymphocyte subtypes (Th17 and Treg cells), microglia M1 marker Iba-1 and astrocyte A1 marker S100β were assayed by ELISA, Th17 cell transcription factor RoRα and Treg cell transcription factors STAT5 and FoxP3^+^ were assayed by q-PCR and ELISA and Treg cell specific marker Helios [24] was assayed by Western Blot (WB). Correlations between depression-like behaviors and two pairs of lymphocyte subtypes, as well as between behaviors and Treg markers or cytokines, were measured. The hypothesis for IL-2 treatment and the contents of the present study are shown in Figure 1.

## 2. Results

### 2.1. Low-Dose IL-2 Attenuated Anxiety- and Depression-Like Behaviors Induced by CUMS

**Figure 2:**
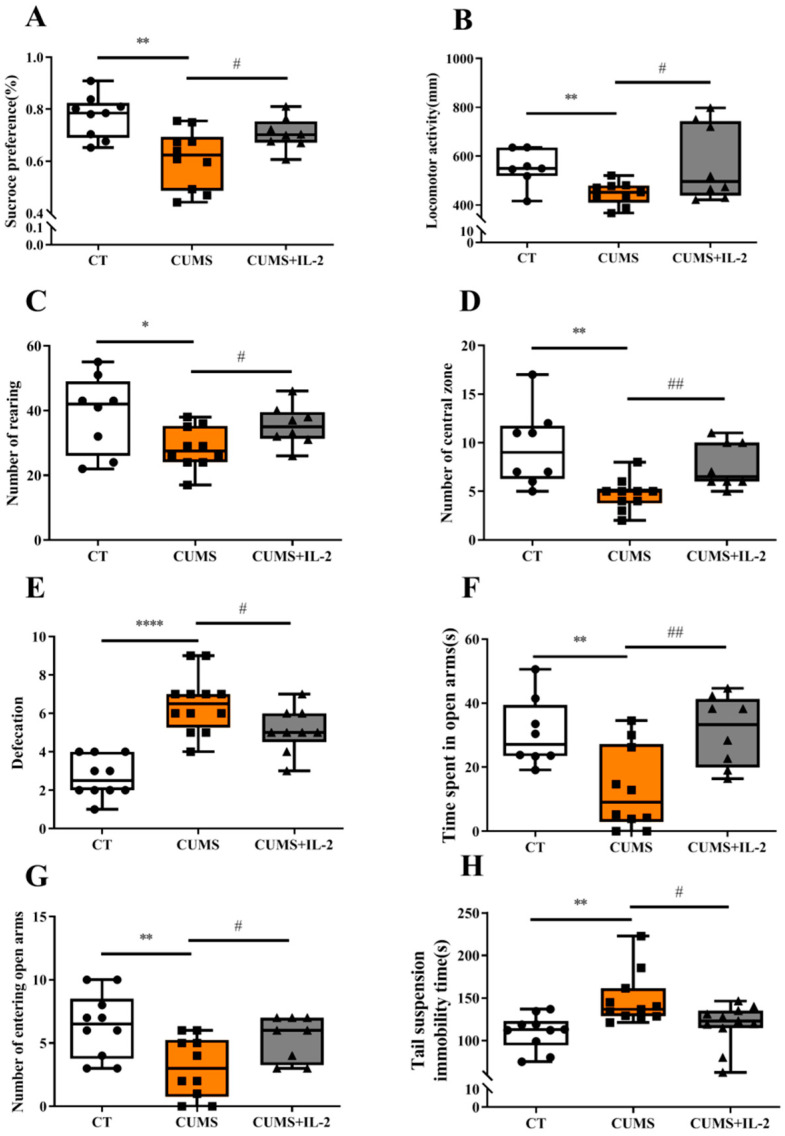
Low-dose IL-2 attenuated anxiety- and depression-like behaviors induced by CUMS exposure. Percentage of sucrose consumption in the sucrose preference test (**A**). Locomotor activity (**B**), number of rearing (**C**), central zone entries (**D**) and defecation (**E**) in the open field test. Time spent (**F**) and number of entries (**G**) into the open arms of the elevated plus maze. Suspension immobility time in the tail suspension test (**H**) (n = 10–12). CT, control; CUMS, CUMS group; CUMS+IL-2, CUMS exposure with IL-2 treatment. **** *p* < 0.0001, ** *p* < 0.01, * *p* < 0.05 vs. CT; # *p* < 0.05, ## *p* < 0.01 vs. CUMS.

As shown in Figure 2, CUMS significantly reduced sucrose solution consumption when compared to the CT group (*p* < 0.01). In the open field test, the locomotor activity (*p* = 0.0027), the number of entries into the central region (*p* = 0.0032) and the rearing number (*p* = 0.0299) were significantly decreased, while the defecation number was increased (*p* < 0.0001) in the CUMS group. In the elevated plus maze test, the number of entries into (*p* = 0.0078) and the time spent (*p* = 0.0069) on the open arms were significantly decreased, while the immobility time was significantly increased (*p* = 0.0022) in the suspension tail test after CUMS exposure.

However, IL-2 treatment significantly increased sucrose solution consumption when compared to the CUMS group (*p* < 0.05). Similarly, the abnormalities of total distance travel (*p* = 0.0342), the number of entries into the central area (*p* = 0.0064), the rearing number (*p* = 0.0338) and the defecation score (*p* = 0.0335) were attenuated by IL-2 treatment. Furthermore, IL-2 treatment exerted effects of anti-anxiety and anti-depression, such as increasing the number of entries into (*p* = 0.039) and the time spent (*p* = 0.0061) on the open arms, as well as decreasing the immobility time in the suspension tail test (*p* = 0.0443).

### 2.2. IL-2 Normalized the Concentration of Monoamine Neurotransmitters and Their Metabolites in the Hippocampus and Amygdala of CUMS Mice

**Figure 3:**
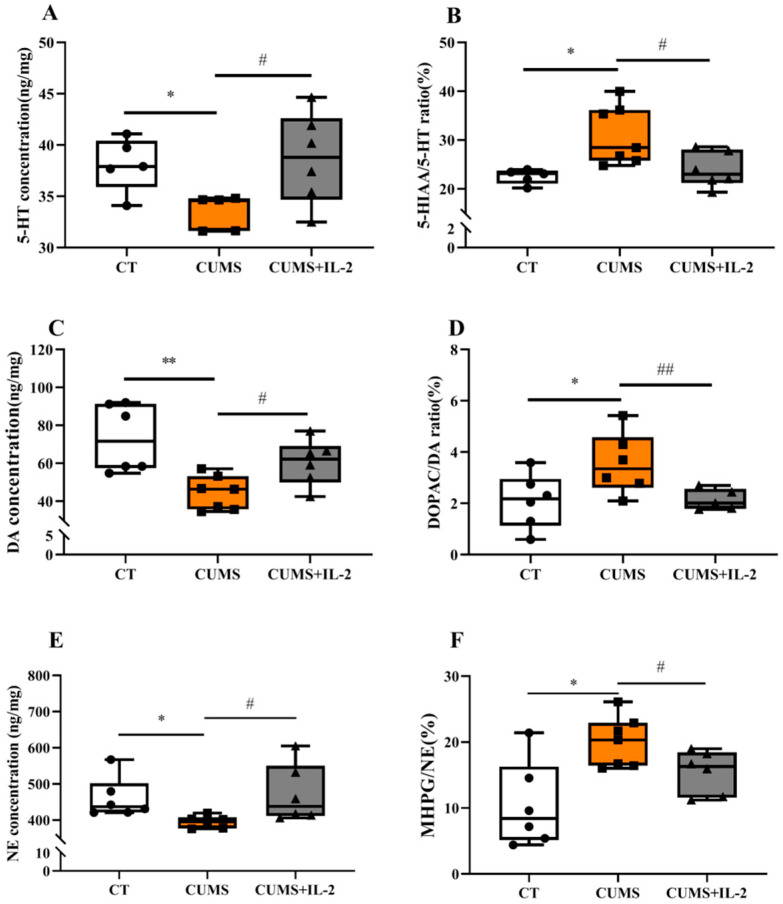
IL-2 normalized the concentration of monoamine neurotransmitters and their metabolites in the hippocampus and amygdala of CUMS mice. 5-HT (**A**), DA (**C**) and their turnovers (**B**,**D**) in the hippocampus; NE (**E**) and its turnover (**F**) in the amygdala (n = 6–8). CT, control; CUMS, CUMS group; CUMS+IL-2, CUMS exposure with IL-2 treatment. ** *p* < 0.01, * *p* < 0.05 vs. CT; # *p* < 0.05, ## *p <* 0.01 vs. CUMS.

Figure 3 shows that the 5-HT (A, *p* = 0.0107) and DA (C, *p* = 0.0030) concentrations were significantly decreased (*p* < 0.05). The ratio of 5-HIAA to 5-HT (B, *p* = 0.0120) and that of DOPAC to DA (D, *p* = 0.0406) was significantly increased in the hippocampus of mice exposed to CUMS when compared with the CT group. CUMS significantly reduced NE (E, *p* = 0.0210) concentration and increased MHPG/NE (F, *p* = 0.0069) in the amygdala (*p* < 0.05). The concentrations of 5-HT (*p* = 0.0366), DA (*p* = 0.0184) and NE (*p* = 0.0428) and the ratios of 5-HIAA/5-HT (*p* = 0.0282), DOPAC/DA (*p* = 0.0340) and MHPG/NE (*p* = 0.0426) were restored by IL-2 treatment.

### 2.3. IL-2 Reversed or Ameliorated CUMS-Induced Changes in Hippocampal Concentrations of Pro- and Anti-Inflammatory Cytokines and IL-17A Receptor Expression

**Figure 4:**
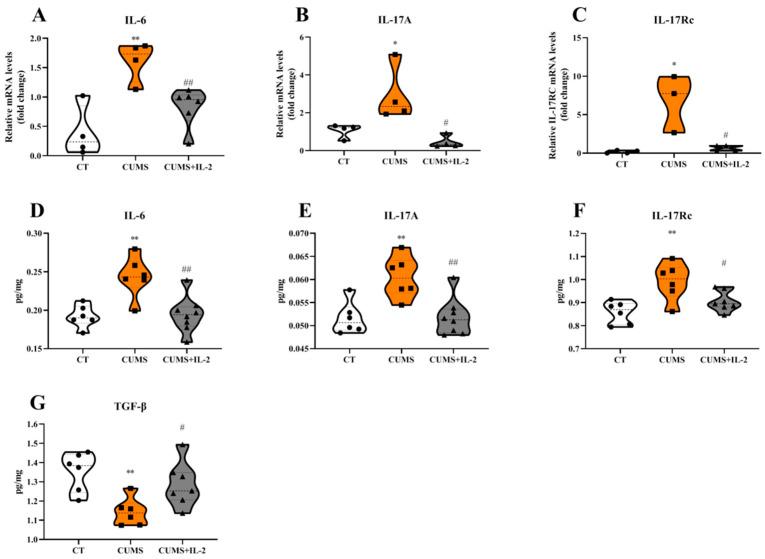
IL-2 reduced the mRNA and protein expression of central pro-inflammatory factors and IL-17A receptor in the mouse hippocampus after CUMS exposure. IL-6 (**A**,**D**), IL-17A (**B**,**E**), IL-17Rc (**C**,**F**) and TGF-β (**G**) (n = 3–8). CT, control group; CUMS, CUMS group; CUMS+IL-2, CUMS exposure with IL-2 treatment. ** *p* < 0.01, * *p* < 0.05 vs. CT; # *p* < 0.05, ## *p* < 0.01 vs. CUMS.

As shown in Figure 4, the mRNA and protein expressions of IL-6 (*p* = 0.00044 and 0.0024), IL-17A (*p* = 0.0492 and 0.0036) and IL-17Rc (*p* = 0.0146 and 0.0032) in the hippocampus were significantly increased, and the protein expression of TGF-β (*p* = 0.0054) was decreased in the CUMS group compared to the CT group. Low-dose IL-2 significantly ameliorated or even reversed CUMS-induced IL-6 (*p* = 0.0066 and 0.0019), IL-17A (*p* = 0.0165 and 0.0026), IL-17Rc (*p* = 0.0198 and 0.0476) and TGF-β (*p* = 0.0483) changes.

### 2.4. IL-2 Restored the mRNA and Protein Expression of Th17 and Treg Cell Transcription Factors in the CUMS Group

**Figure 5:**
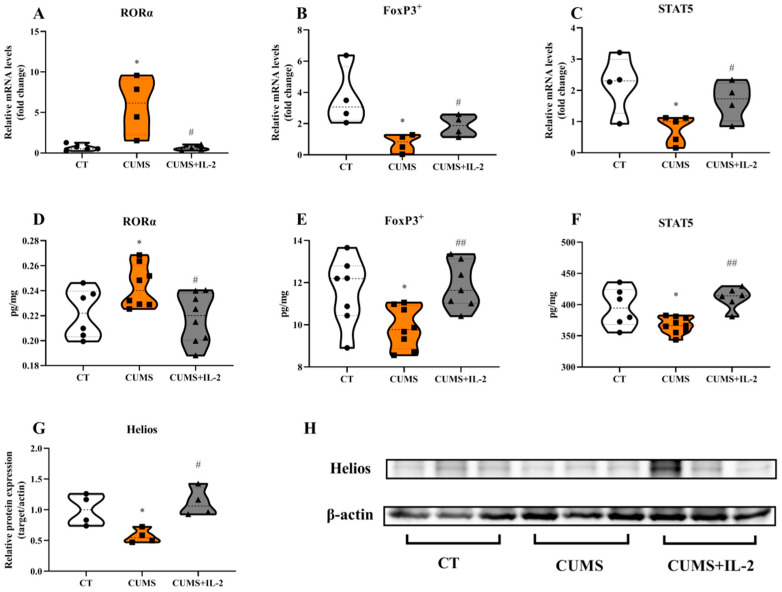
IL-2 restored the mRNA and protein expression of Th17 and Treg cell transcription factors in the hippocampus of CUMS mice. Th17 transcription factor RoRα (**A**,**D**), Treg transcription factor FoxP3^+^ (**B**,**E**), STAT5 (**C**,**F**) and Helios (**G**,**H**) in the hippocampus (n = 4–8). CT, control group; CUMS, CUMS group; CUMS+IL-2, CUMS exposure with IL-2 treatment. * *p* < 0.05 vs. CT; # *p* < 0.05, ## *p* < 0.01 vs. CUMS.

Figure 5 shows that CUMS significantly increased the mRNA and protein expression of the Th17 cell transcription factor RoRα in the mouse hippocampus when compared with the CT group (*p* = 0.0136 and 0.0478). However, the Treg cell transcription factor FoxP3^+^ (*p* = 0.0272 and 0.0245), STAT5 (*p* = 0.0191 and 0.0423) and Helios (*p* = 0.0405) were significantly decreased after being exposed to CUMS when compared with the CT group. RoRα (*p* = 0.0267 and 0.0150), FoxP3^+^ (*p* = 0.0440 and 0.0027), STAT5 (*p* = 0.0404 and 0.0020) and Helios (*p* = 0.0118) were significantly normalized by IL-2 in the CUMS group (*p* < 0.05, *p* < 0.01).

### 2.5. IL-2 Inhibited the Activation of M1 and A1 Phenotypes and the IDO Enzyme Induced by CUMS

**Figure 6:**
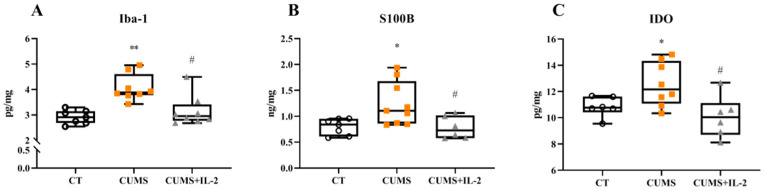
IL-2 attenuated the increased expression of microglia M1 phenotype marker Iba-1 (**A**), astrocyte A1 phenotype marker S100β (**B**) and IDO enzyme (**C**) in the hippocampus in the CUMS group (n = 6–8). CT, control group; CUMS, CUMS group; CUMS+IL-2, CUMS exposure with IL-2 treatment. ** *p* < 0.01, * *p* < 0.05 vs. CT; # *p* < 0.05 vs. CUMS.

The protein expressions of microglia M1 phenotype marker Iba-1 (*p* = 0.0016), astrocyte A1 phenotype marker S100β (*p* = 0.0241) and IDO enzyme (*p* = 0.0409) were significantly increased in the mouse hippocampus upon CUMS exposure when compared with the CT group, while low-dose IL-2 significantly inhibited Iba-1 (*p* = 0.0139), S100β (*p* = 0.0255) and IDO enzyme (*p* = 0.0160) changes (Figure 6).

### 2.6. IL-2 Did Not Attenuate Increased CORT Concentration in the CUMS Group

**Figure 7:**
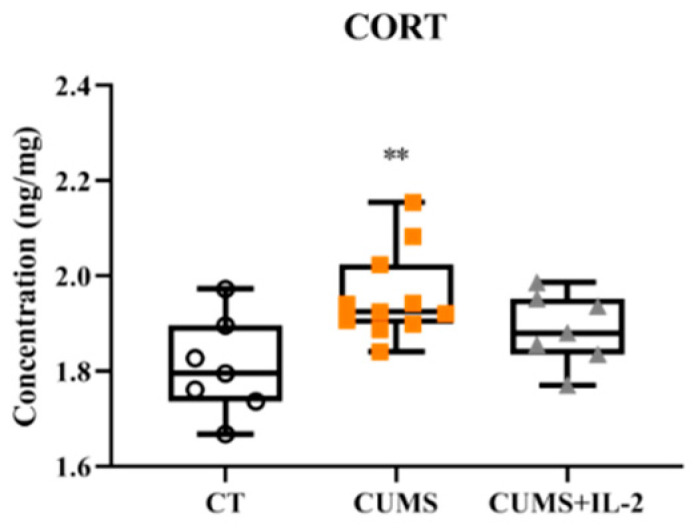
IL-2 did not significantly decrease the corticosterone concentration in the serum of CUMS mice (n = 6–8). CT, control group; CUMS, CUMS group; CUMS+IL-2, CUMS exposure with IL-2 treatment. ** *p* < 0.01 vs. CT.

As shown in Figure 7, the CORT concentration was significantly increased in the serum after CUMS exposure when compared with the CT group (*p* = 0.0074). However, low-dose IL-2 did not significantly attenuate the CORT concentration (*p* = 0.1206).

### 2.7. The Correlations among Different Parameters

**Figure 8:**
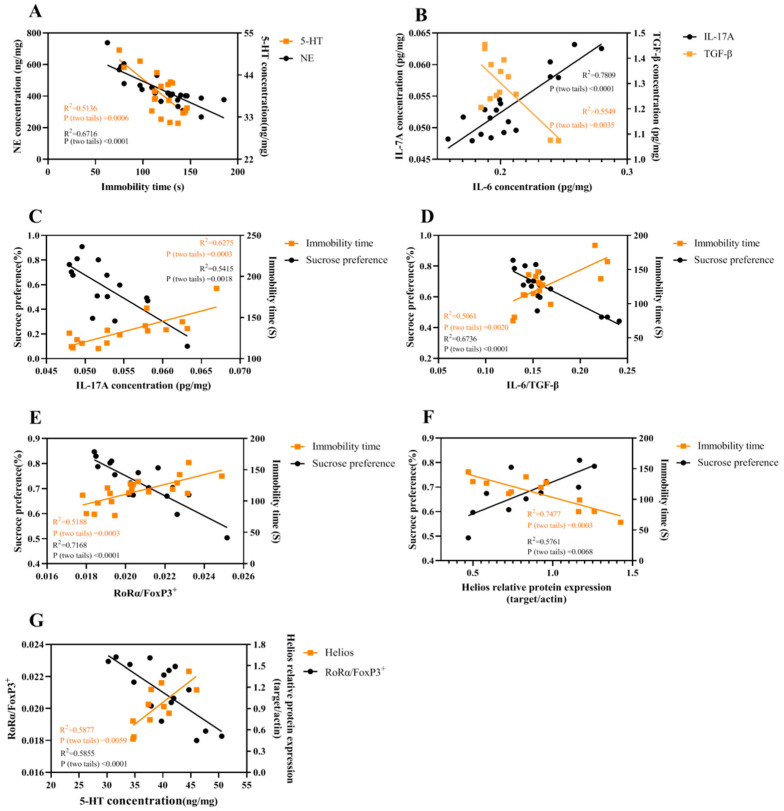
The correlations among different parameters were analyzed. Correlations between immobility time and NE or 5-HT (**A**), between IL-6 and TGF-β or IL-17A (**B**), between IL-17A and behaviors (**C**), between IL-6/TGFβ and behaviors (**D**), between Th17/Treg and behaviors (**E**), between Helios and behaviors (**F**) and between 5-HT and Th17/Treg or Helios (**G**).

In Figure 8A, the immobility time was correlated with NE (R^2^ = 0.6716, *p* < 0.0001) and 5-HT (R^2^ = 0.5136, *p* = 0.0006); the correlation between IL-6 and TGF-β (R^2^ = 0.5549, *p* = 0.0035) or IL-17A (R^2^ = 0.7809, *p* < 0.0001) is shown in Figure 8B; Figure 8C–F shows that the behaviors (sucrose preference and immobility time) were correlated with IL-17A (R^2^ = 0.5415, *p* = 0.0018; R^2^ = 0.6275, *p* = 0.0003;), IL-6/TGF-β (R^2^ = 0.6736, *p* < 0.0001; R^2^ = 0.5061, *p* = 0.0020), RoRα/FoxP3^+^ (R^2^ = 0.7168, *p* < 0.0001; R^2^ = 0.5188, *p* = 0.0003) and Helios (R^2^ = 0.5761, *p* = 0.0068; R^2^ = 0.7477, *p* = 0.0003), respectively. The correlation between 5-HT and RoRα/FoxP3^+^ (R^2^ = 0.5855, *p* < 0.0001) or Helios (R^2^ = 0.5877, *p* = 0.0059) is shown in Figure 8G. However, no significant correlation between NE and RoRα/FoxP3^+^ or Helios (R^2^ = 0.3636; R^2^ = 0.0245) was found.

## 3. Discussion

This study, for the first time, demonstrated that CUMS significantly resulted in imbalances between IL-6 and TGF-β and between Th17 and Treg in the brain, which were restored by low-dose IL-2 treatment. In contrast, IL-2 treatment attenuated and even reversed depression- and anxiety-like behaviors by normalizing the deficiency of monoamine neurotransmitters and the increase in neurotransmitter metabolism in the hippocampus and amygdala. The mechanisms by which IL-2 successfully treated CUMS-induced depressive symptoms and underlined neuroimmunological changes are discussed below.

### 3.1. The Imbalance between IL-6 and TGF-β in the Hippocampus of CUMS Mice

Microglia are macrophages in the brain (the same as astrocytes) which are activated from the resting state to the M1 and A1 phenotype, respectively, by peripheral inflammatory signals, oxidants and chronic stress [25]. Microglia M1 and astrocyte A1 polarization further releases various proinflammatory factors, such as IL-1 and IL-6, which cause neuroinflammation [26,27]. By contrast, microglia M2 and astrocyte A2 phenotypes can release anti-inflammatory cytokines and neurotrophins, such as IL-10, TGF-β and BDNF [28,29]. Our team and others have recently reported that the CUMS-activated HPA axis and increased CORT concentration can trigger microglia M1 marker Iba-1 expression and decreased TGF-β concentration in the brain [30,31,32,33]. Furthermore, an over-activated M1 phenotype led to the dysfunction of monoamine neurotransmitters through increasing IDO activity [7], thereby causing depression- and anxiety-like behaviors. Astrocyte A1 was found to express S100β, which promotes the expression of inducible nitric oxide synthase or pro-inflammatory cytokines and exhibits detrimental effects on neurons [34]. Significantly increased S100β was found in the hippocampus of stressed mice, indicating an activation of A1 after CUMS exposure, which was similar to what Horiguchi reported [35]. Thus, the present study confirmed these findings and further demonstrated the CUMS-induced imbalance between IL-6 and TGF-β through the microglia M1 and A1 activation.

### 3.2. The Imbalance between Th17 and Treg Occurred in the Hippocampus of CUMS Mice

Th17 and Treg cells are a pair of interacting lymphocyte subtypes. Under the stimulation and activation of antigens, Th17 cells secret proinflammatory factors—for example IL-17A, which participates in the peripheral inflammation of autoimmune diseases. For example, psoriasis, an autoimmune disease is caused by the excessive secretion of IL-17. Those patients are often accompanied by depressive symptoms. Furthermore, treatment with the IL-17 inhibitor not only significantly attenuated psoriatic pathological features but also markedly reduced depressive symptoms [31]. In contrast to Th17, Treg cells are the main executor of immune tolerance [36]. Treg transcription factor FoxP3^+^ suppresses the expression of transcription factor RoRα, which inhibits the differentiation and proliferation of Th17 cells [37]. Moreover, a recent study found that Treg has a specific transcription factor, Helios, that contributes to stabilizing Treg survival through STAT5 activation and IL-2 response [24]. The present study found that CUMS markedly increased RoRα and decreased FoxP3^+^ and STAT5 at both levels of mRNA and protein expression, as well as Helios at the protein expression level, which indicates an imbalance between two lymphocyte subtypes.

The imbalance between Th17 and Treg may result from the imbalance between IL-6 and TGF-β. Many studies have shown that IL-6 can activate transcription factor STAT3 by binding with IL-6R in the Th17 cells [38], while TGF-β can increase transcription factor FoxP3^+^ by binding with TGF-βR in Treg cells [39,40]. The other possibility is that CUMS causes an imbalance between T17 and Treg subtypes, which may be directly induced by microglia M1 response.

The activation of microglia can release T lymphocyte chemokines, which promotes the differentiation and migration of T cells into the center [41]. Therefore, the findings of the up-regulation of the Th17-related marker RoRa and the down-regulation of the Treg marker FoxP3^+^ from the present study may result from the activation of microglia M1 phenotype after CUMS exposure.

Since it is unclear whether CUMS aggravates neuroinflammation through the imbalance between Th17 and Treg, the present study further evaluated the mRNA and protein expression of IL-17A and its receptor IL-17Rc in a model of depression. IL-17Rc, a specific receptor of IL-17A, is expressed in microglia and astrocytes [11]. In the current study, CUMS significantly increased the mRNA and protein expression of Iba-1, S100β, IL-17A and IL-17Rc in the hippocampus, while it decreased FoxP3^+^. Thus, the excessive release of IL-17A and the binding with IL-17Rc in microglia and astrocytes may further trigger neuroinflammation, thereby causing depression.

### 3.3. Low-Dose IL-2 Improved Depression-Like Behavior and Neuropathological Changes through Restoring the Balance between Two Pairs of Lymphocyte Subtypes

According to the hypothesis, the present study, for the first time, demonstrated that the intraperitoneal injection of low-dose IL-2 markedly treated depression- and anxiety-like behaviors by restoring the imbalance between Treg and Th17 in the hippocampus of CUMS mice. As we introduced above, IL-2 alleviated depressive behaviors and symptoms through activating Treg cells. IL-2, an important part of Treg cell signal transduction, transmits signals to up-regulate the Treg cell transcription factor FoxP3^+^ after binding to IL-2R [19]. Indeed, the present study showed that the effect of IL-2 on the Th17 and Treg balance was associated with the increase in Foxp3^+^, STAT5 and Helios expression and the decrease in RoRα expression, which may result in the balance between IL-6 and TGF-β, and it attenuated or even reversed M1 phenotype microglia and A1 phenotype astrocyte activation. More importantly, IL-2 reduced Th17 differentiation, consequently decreasing IL-17A secretion, and alleviated the expression of IL-17Rc in microglia and astrocytes, which inhibited the activation of microglia M1 and astrocyte A1. It has been extensively reported that the activated M1 phenotype can enhance IDO enzyme activity. Consistent with this, the present study found that the suppression of M1 response by IL-2 can also decrease IDO enzyme activity, which restored the deficiency of monoamine neurotransmitter levels and eventually attenuated depression-like behaviors. However, we found that CORT concentration was not significantly decreased after IL-2 treatment. A similar result was reported by Anisman’s team [42]. It is possible that the IL-2 effects on depression-like changes were not via CORT or that the treatment duration was not enough to significantly change CORT.

After analyzing several correlations, the present study demonstrated that depression-like behaviors were negatively correlated with 5-HT and NE, which was consistent with the classic monoamine deficient hypothesis of depression. More importantly, IL-6 was correlated with IL-17A and TGF-β, indicating that IL-6 not only inhibited TGF-β expression but also promoted IL-17A release. IL-17A and IL-6/TGF-β were negatively correlated with sucrose preference and positively correlated with immobility time. This fits our hypothesis that increased IL-17A and IL-6/TGF-β might contribute to depression-like behaviors. Moreover, the Th17/Treg transcription factor RoRα/FoxP3^+^ was significantly and negatively correlated with 5-HT contents, while it was positively correlated with depression-like behaviors. Conversely, the Treg transcription factor Helios was positively correlated with 5-HT and negatively correlated with depression-like behaviors. These findings further demonstrated that the Th17/Treg imbalance toward Th17 may induce 5-HT deficiency, thereby causing depression. Furthermore, Th17-induced changes were alleviated by enhanced Treg differentiation, such as the up-regulated Helios after IL-2 treatment.

In conclusion, we determined that the restoration of balances between IL-6 and TGF-β and between Th17 and Treg could significantly inhibit proinflammatory cytokine concentrations and, more specifically, reduce the IL-17A concentration. Then, the reduction in IL-17A inhibited its receptor expression, prevented the over-activation of microglia and astrocyte inflammatory phenotypes and reduced IDO enzyme concentrations, which normalized the neurotransmitter concentration and metabolism in the hippocampus and amygdala, thereby improving the depression- and anxiety-like behaviors induced by CUMS. Our correlation analysis findings further indicated that these two balances were correlated with behavioral deficits and recovery—especially, Th17 and Treg exhibited opposite correlations with 5-HT, respectively. Therefore, the balance between two pairs of lymphocytes may be considered as a potential target for depression treatments.

The limitation of this study was the localization of the Th17- and Treg-related markers in the brain by immunohistochemistry (IHC). This limitation should be resolved in a future study.

## 4. Materials and Methods

### 4.1. Materials

IL-2 (Beyotime Biotechnology Co., Ltd., Shanghai, China) was dissolved into sterile physiological saline at a dose of 1500UI/100 μL/ day [43].

First, 6–8 week-old male ICR mice were purchased from Huafukang Biotechnology Co., Ltd. (Beijing, China), with the production license No. SCXK (Yue) 20110015. The mice were housed as three per cage under SPF standard room conditions (12 h light/dark cycle, 23 ± 2 °C, relative humidity of 50%–60%, free access to food and water). The experimental protocol for animals was in accordance with the relevant regulations and was approved by the Animal Care and Use Committee of Guangdong Ocean University, China (IACUC-20160922-037). After 14 days of adaptation to the new conditions, the mice were divided into three groups (n = 12): the control (CT), CUMS and CUMS + IL-2 groups.

### 4.2. Experimental Procedure

Except for the CT group, the mice were randomly exposed to two different types of stressors every day, with no stressor repeated within 3 days, as in our previous study, for 42 days [43]. Strategies are shown in Table 1. Seven days before the behavioral tests, a sterile 1 mL syringe with IL-2 was inserted to a depth of 0.5 cm and at a 45° angle in the lower right abdomen of the mice [44] and daily intraperitoneally injected for 12 days until the mice were sacrificed. Other mice were injected with sterile saline. Since our previous studies and other previous studies did not find that low-dose IL-2 significantly affects control animal behavior, immune function and neurotransmitter concentrations [14,20], we left out the CT+IL-2 group in the interest of saving animal lives.

**Figure 9:**
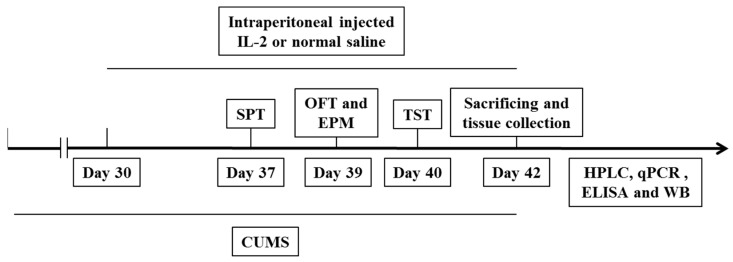
Experimental procedures. CUMS, chronic unpredictable mild stress; SPT, sucrose preference; OFT, open field; EPM, elevated plus maze; TST, tail suspension tests; HPLC, high-performance liquid chromatography; qPCR, quantitative real-time polymerase chain reaction; ELISA, Enzyme-linked immunosorbent assay; WB, Western blot.

After behavioral tests, the animals were decapitated. Blood samples were collected, and then the brain tissues were carefully dissected on ice and frozen in liquid nitrogen rapidly. The samples were stored at −80 °C until further measurements (Figure 9).

### 4.3. Behavioral Tests

#### 4.3.1. Sucrose Preference Test

The mice were trained to consume sucrose solution prior to the CUMS procedure. During the training period, two bottles of 1% (*m*/*v*) sucrose solution were given to the mice at the first 24 h. One of the bottles was replaced by pure water on the following day. Then, food and water deprivation was carried out for 24 h before the sucrose preference test. After deprivation, every mouse was separated and could choose to drink from a 1% sucrose solution or a tap water bottle for 4 h. The sucrose preference rate (%) is calculated based on the ratio of the sucrose solution consumed and the total liquid consumed [45].

#### 4.3.2. Open Field Test

The mice were placed in an open field apparatus (40 cm in length, width and height, with a white floor) facing the wall. A central area (8 cm in length and width) was drawn with the Super Maze behavior analysis system (Xinruan Information Technology Co., Ltd., Shanghai, China). A 60 watt bulb was positioned 90 cm above the center of the apparatus. The rearing number times, the locomotor activity total distance traveled, the center area entry number times and the defecation score were recorded within 5 min and analyzed by the behavior analysis system. At the end of each test, the apparatus was cleaned thoroughly with 75% alcohol and water [20].

#### 4.3.3. Elevated Plus-Maze Test

The elevated plus-maze is comprised of two opposite open arms, two enclosed arms and a central area, with a height of 60 cm from the ground. The arms of the maze are 25 cm long and 5 cm wide; the two enclosed arms have sides that are 10 cm high. Lighting was provided by a 40 watt bulb above the maze. During the experiment, each mouse was placed in the center area and faced the open arm. The behavior analysis system (Shanghai Xinruan Information Technology Co., Ltd., Shanghai, China) was used to record the entries into and time spent on the open arms within 5 min [20].

#### 4.3.4. Tail Suspension Test

Each mouse was suspended by its tail with adhesive tape 60 cm above the ground. The immobility time spent was recorded within 5 min [46].

### 4.4. HPLC Analysis of Neurotransmitters and Metabolites in the Hippocampus and Amygdala

Hippocampal and amygdala tissues were homogenized in 0.60 M perchloric acid containing 50 mM Na_2_EDTA, centrifuged at 14,000 r/ min for 15 min, isolated with supernatant mixed with perchloric acid precipitant and centrifuged again for 15 min. The supernatants were filtrated by 0.45 μm membrane for the analysis. For the HPLC analysis, 20 μL supernatant was injected into the HPLC system with a fluorescence detector (Agelent, Santa Clara, CA, USA). The neurotransmitters and their metabolites in samples were separated by a C18 reverse-phase column (4.6 × 150 mm) (Agelent, Santa Clara, CA, USA) with citrate solution. The citrate solution was prepared as follows: 0.1 M sodium acetate was mixed with 0.1 M citric acid in the ratio of 10:9 and supplemented with sodium octane sulfonate (100 mg/L). The ratio of the citrate solution and methanol was regulated at 87:13 in the system. Norepinephrine (NE), methoxy hydroxyphenylethylene glycol (MHPG), dopamine (DA), dihydroxyphenylacetic acid (DOPAC), 5-hydroxytryptamine (5-HT) and 5-hydroxyindoleacetic acid (5-HIAA) were analyzed [47].

### 4.5. Quantitative Real-Time PCR

The total RNA was extracted from the hippocampus brain area with Trizol reagents (Qiyun Biotechnology Co., Ltd., Guangzhou, China), following the manufacturer’s protocol. The concentration of RNA was quantified by ultraviolet spectrophotometry (DeNovix Co., Ltd., Delaware, America) at 260/280 nm. Complementary DNA (cDNA) was synthesized from 1 μg total RNA using the HiScript ^®^ II Q Select RT SuperMix kit (Vazyme Biotech Co., Ltd., Nanjing, China). PCR amplifications were performed on a ABI7500 Real-Time PCR Detection System (Thermo Fisher Scientific Co., Ltd., Shanghai, China) using the SYBR qPCR Master Mix kit (Vazyme Biotech Co., Ltd., Nanjing, China) under the following conditions: initial activation at 95 °C for 30 s, followed by 40 cycles of amplification (95 °C for 5 s, 60 °C for 30 s) [48]. The fold change was calculated using the 2-ddCt method, normalized against the internal control, GAPDH. The primer sequences for the target genes and GAPDH are listed in Table 2.

### 4.6. ELISA Assays for Cytokines, Their Receptor and Microglia Markers

Hippocampal tissues were weighed and ground after adding PBS in a 1 mg: 9 μL proportion. The levels of IL-6, TGF-β, FoxP3+, RoRα, STAT5, IL-17A, IL-17Rc, S100β, Iba-1, IDO enzyme and CORT were measured using commercially available enzyme-linked immunosorbent assay (ELISA) kits (Dogesce Biotechnology Co., Ltd., Beijing, China), according to the manufacturer’ s protocol.

### 4.7. Western Blot

The proteins were extracted from the hippocampal tissues, and the total protein concentration in the supernatants was measured with a BCA kit (Beyotime technology, China). The samples were separated by 10% SDS-PAGE, transferred onto PVDF membranes and then blocked with 5% skim milk in TBS-T. The membranes were incubated with the primary antibodies anti-Helios (SC-390357 HRP) and anti-β-actin (SC-47778 HRP) overnight at 4 °C. The membranes were scanned by an imaging system (Azure Biosystems C600, America) and then quantified using Image J software (National Institutes of Health, USA).

### 4.8. Statistical Analysis

The results are expressed as the mean ± standard error of the mean (SEM). The data were analyzed by Graphpad Prism 8.0. Statistical analyses were performed by one-way analysis of variance (ANOVA), followed by Tukey’s post hoc test as multiple comparisons between groups. The correlations between several pairs of parameters were analyzed by Pearson correlation analysis (R^2^ > 0.5). Significance was indicated at a *p*-value < 0.05.

## Figures and Tables

**Table 1:** Chronic unpredictable mild stress procedure. Dx (x = 1, 2, … 45) indicates day X.

Stressor	Days
Shaker for 1.5 h	D4,D8,D11,D17,D23,D30,D36,D41
4 °C environment for 1 h	D1,D5,D9,D14,D18,D24,D29,D35,D40
Swimming at 18 °C for 10 min	D2,D6,D13,D20,D26,D33,D38
Crowded environment for 3 h	D3,D7,D10,D15,D21,D27,D32,D36,D41
Tilt the cage 45° for 12 h	D3,D7,D13,D16,D22,D26,D31,D37
Illumination for 24 h	D1,D9,D12,D18,D22,D27,D31,D34,D37,D40
Strobe lamp	D10,D16,D20,D25,D30,D33,D42
Peculiar smell	D12,D21,D29,D35,D42
Lack of water	D2,D6,D11,D15,D19,D23,D28,D34,D39
Lack of feed	D5,D14,D19,D24,D28,D39
Wet bedding	D4,D8,D17,D25,D32,D38

**Table 2:** Primer sequences of quantitative PCR.

Mouse Genes	Primer Sequences
IL-6 F	5′-GAGGATACCACTCCCACAGACC-3′ 5′-AAGTGCATCATCGTTGTTCATACA-3′
IL-6 R	
IL-17A F	5′-CCCTCAGACTACCTCAACCGTTC-3′ 5′-TTCATGTGGTGGTCCAGCTTTC-3′
IL-17A R	
IL-17RA F	5′-AGTTCCAGTTTCTGTCCATGC-3′ 5′-TGGATTTGTGGTTTGGGTC-3′
IL-17RA R	
IL-17RC F	5′-GCAGAGCCTGAAGAAGCTG-3′ 5′-CCCAAGACTAGCCTCGAAAC-3′
IL-17RC R	
STAT3 F	5′-CTACAGTGACAGCTTCCCAAT-3′ 5′-TTGGCTTCTCAAGATACCTGC-3′
STAT3 R	
RoRa F	5′-TCACCTCTCTGCTTGTTCTG-3′ 5′-GCTTCTTCCCCTACTGTTCCTT-3′
RoRa R	
STAT5 F	5′-CAAGCAAGTGGTCCCTGAGT-3′ 5′-CATTGGTCGGCGTAAGAGTT-3′
STAT5 R	
FoxP3^+^ F	5′-GGCCCTTCTCCAGGACAGA-3′ 5′-GCTGATCATGGCTGGGTTGT-3′
FoxP3^+^ R	
GAPDH F	5′-GAAGGTGAAGGTCGGAGTC-3′ 5′-GAAGATGGTGATGGGATTTC-3′
GAPDH R	

## Data Availability

Not applicable.

## References

[1] Dong Y.F., Wang X.Y., Zhou Y., Zheng Q.M., Chen Z., Zhang H., Sun Z.L., Xu G.H., Hu G. (2020). Hypothalamus-pituitary-adrenal axis imbalance and inflammation contribute to sex differences in separation- and restraint-induced depression. Horm. Behav..

[2] Delgado P.L. (2000). Depression: The case for a monoamine deficiency. J. Clin. Psychiatry.

[3] Burrows K., Stewart J.L., Kuplicki R., Figueroa-Hall L., Spechler P.A., Zheng H. (2021). Elevated peripheral inflammation is associated with attenuated striatal reward anticipation in major depressive disorder. Brain Behav. Immun..

[4] Song C. (2002). The effect of thymectomy and IL-1 on memory: Implications for the relationship between immunity and depression. Brain Behav. Immun..

[5] Mello C., Swain M.G. (2016). Immune-to-Brain Communication Pathways in Inflammation-Associated Sickness and Depression. Curr. Top. Behav. Neurosci..

[6] Wang Q., Jie W., Liu J.H., Yang J.M., Gao T.M. (2017). An astroglial basis of major depressive disorder? An overview. Glia.

[7] Da Silva Dias I.C., Carabelli B., Ishii D.K., de Morais H., de Carvalho M.C., Rizzo de Souza L.E., Zanata S.M., Brandão M.L., Cunha T.M., Ferraz A.C. (2016). Indoleamine-2, 3-Dioxygenase/Kynurenine Pathway as a Potential Pharmacological Target to Treat Depression Associated with Diabetes. Mol. Neurobiol..

[8] Wang D., Huang S., Yuan X., Liang J., Xu R., Yao G., Sun L. (2017). The regulation of the Treg/Th17 balance by mesenchymal stem cells in human systemic lupus erythematosus. Cell. Mol. Immunol..

[9] Ding H., Dai Y., Lei Y., Wang Z., Liu D., Li R., Hu Y. (2019). Upregulation of CD81 in trophoblasts induces an imbalance of Treg/Th17 cells by promoting IL-6 expression in preeclampsia. Cell. Mol. Immunol..

[10] Mantovani A., Sica A., Sozzani S., Allavena P., Vecchi A., Locati M. (2004). The chemokine system in diverse forms of macrophage activation and polarization. Trends Immunol..

[11] Nichols J.R., Aldrich A.L., Mariani M.M., Vidlak D., Esen N., Kielian T. (2009). TLR2 deficiency leads to increased Th17 infiltrates in experimental brain abscesses. J. Immunol..

[12] Waisman A., Hauptmann J., Regen T. (2015). The role of IL-17 in CNS diseases. Acta Neuropathol..

[13] Liblau R.S. (2017). Endothelial cells and lymphatics at the interface between the immune and central nervous systems: Implications for multiple sclerosis. Curr. Opin. Neurol..

[14] Song C., Merali Z., Anisman H. (1999). Variations of nucleus accumbens dopamine and serotonin following systemic interleukin-1, interleukin-2 or interleukin-6 treatment. Neuroscience.

[15] Fakhri H., Lang F., Lang U.E., Ricken R. (2021). Faster speed of onset of the depressive episode is associated with lower cytokine serum levels (IL-2, -4, -6, -10, TNF-α and IFN-γ) in patients with major depression. J. Psychiatr. Res..

[16] Darko D.F., Gillin J.C., Risch S.C., Bulloch K., Golshan S., Tasevska Z., Hamburger R.N. (1989). Mitogen-stimulated lymphocyte proliferation and pituitary hormones in major depression. Biol. Psychiatry.

[17] Fontenot J.D., Rasmussen J.P., Gavin M.A., Rudensky A.Y. (2005). A function for interleukin 2 in Foxp3-expressing regulatory T cells. Nat. Immunol..

[18] Lee H., Son Y.S., Lee M.O., Ryu J.W., Park K., Kwon O., Son M.Y. (2020). Low-dose interleukin-2 alleviates dextran sodium sulfate-induced colitis in mice by recovering intestinal integrity and inhibiting AKT-dependent pathways. Theranostics.

[19] Sharabi A., Li H., Kasper I.R., Pan W., Meidan E., Tsokos M.G., Tsokos G.C. (2019). PP2A enables IL-2 signaling by preserving IL-2Rβ chain expression during Treg development. JCI Insight.

[20] Song C., Leonard B.E. (1995). Interleukin-2-induced changes in behavioural, neurotransmitter, and immunological parameters in the olfactory bulbectomized rat. Neuroimmunomodulation.

[21] Smith R.S. (1991). The macrophage theory of depression. Med. Hypotheses.

[22] Lutter M., Sakata I., Osborne L.S., Rovinsky S.A., Anderson J.G., Jung S., Birnbaum S., Yanagisawa M., Elmquist J.K., Nestler E.J. (2008). The orexigenic hormone ghrelin defends against depressive symptoms of chronic stress. Nat. Neurosci..

[23] Stone E.A., Quartermain D. (1999). Alpha-1-noradrenergic neurotransmission, corticosterone, and behavioral depression. Biol. Psychiatry.

[24] Kim H.J., Barnitz R.A., Kreslavsky T., Brown F.D., Moffett H., Lemieux M.E., Kaygusuz Y., Meissner T., Holderried T.A., Chan S. (2015). Stable inhibitory activity of regulatory T cells requires the transcription factor Helios. Science.

[25] Liddelow S.A., Marsh S.E., Stevens B. (2020). Microglia and Astrocytes in Disease: Dynamic Duo or Partners in Crime?. Trends Immunol..

[26] West P.K., Viengkhou B., Campbell I.L., Hofer M.J. (2019). Microglia responses to interleukin-6 and type I interferons in neuroinflammatory disease. Glia.

[27] Roberts A.J., Khom S., Bajo M., Vlkolinsky R., Polis I., Cates-Gatto C., Gruol D.L. (2019). Increased IL-6 expression in astrocytes is associated with emotionality, alterations in central amygdala GABAergic transmission, and excitability during alcohol withdrawal. Brain Behav. Immun..

[28] Gu M., Li X., Yan L., Zhang Y., Yang L., Li S., Song C. (2021). Endogenous ω-3 fatty acids in Fat-1 mice attenuated depression-like behaviors, spatial memory impairment and relevant changes induced by olfactory bulbectomy. Prostaglandins Leukot. Essent. Fat. Acids.

[29] Liu Q., Zhang Y.L., Liu S., Liu Y., Yang X., Liu G., Ma J. (2019). Cathepsin C promotes microglia M1 polarization and aggravates neuroinflammation via activation of Ca-dependent PKC/p38MAPK/NF-κB pathway. J. Neuroinflammation.

[30] Li C., Wu H., Sen Ta Na H., Wang L., Zhong C., Deng B., Liu C., Bao H., Sang H., Hou L. (2022). Neuronal-Microglial Liver X receptor β Activating Decrease Neuroinflammation and Chronic Stress-induced Depression-Related Behavior in Mice. Brain Res..

[31] Ye Y., Wang G., Wang H., Wang X. (2011). Brain-derived neurotrophic factor (BDNF) infusion restored astrocytic plasticity in the hippocampus of a rat model of depression. Neurosci. Lett..

[32] Duan C.M., Zhang J.R., Wan T.F., Wang Y., Chen H.S., Liu L. (2020). SRT2104 attenuates chronic unpredictable mild stress-induced depressive-like behaviors and imbalance between microglial M1 and M2 phenotypes in the mice. Behav. Brain Res..

[33] Aguilar C., Castro O., Ortega E.M., Maldonado-García C., Cruz J.S., Pérez-Montesinos G., Bonifaz L.C. (2020). Association of Pathogenic Th17 Cells with the Disease Severity and Its Potential Implication for Biological Treatment Selection in Psoriasis Patients. Mediators Inflamm..

[34] Tateishi N., Shimoda T., Yada N., Shinagawa R., Kagamiishi Y. (2006). S100B: Astrocyte specific protein. Nihon Shinkei Seishin Yakurigaku Zasshi.

[35] Horiguchi K., Higuchi M., Yoshida S., Nakakura T., Tateno K., Hasegawa R., Kato Y. (2014). Proton receptor GPR68 expression in dendritic-cell-like S100β-positive cells of rat anterior pituitary gland: GPR68 induces interleukin-6 gene expression in extracellular acidification. Cell Tissue Res..

[36] Du J., Huang C., Zhou B., Ziegler S.F. (2008). Isoform-specific inhibition of ROR alpha-mediated transcriptional activation by human FOXP3. J. Immunol..

[37] Ihle J.N., Kerr I.M. (1995). Jaks and Stats in signaling by the cytokine receptor superfamily. Trends Genet..

[38] Zhou L., Lopes J.E., Chong M.M.W., Ivanov I.I., Min R., Victora G.D., Littman D.R. (2008). TGF-beta-induced Foxp3 inhibits T (H) 17 cell differentiation by antagonizing RORgammat function. Nature.

[39] Freudenberg K., Lindner N., Dohnke S., Garbe A.I., Schallenberg S., Kretschmer K. (2018). Critical Role of TGF-β and IL-2 Receptor Signaling in Foxp3 Induction by an Inhibitor of DNA Methylation. Front. Immunol..

[40] Yu W.B., Wang Q., Chen S. (2019). The therapeutic potential of ginkgolide K in experimental autoimmune encephalomyelitis via peripheral immunomodulation. Int. Immunopharmacol..

[41] Klatzmann D., Abbas A.K. (2015). The promise of low-dose interleukin-2 therapy for autoimmune and inflammatory diseases. Nat. Rev. Immunol..

[42] Sudom K., Turrin N.P., Hayley S., Anisman H. (2004). Influence of chronic interleukin-2 infusion and stressors on sickness behaviors and neurochemical change in mice. Neuroimmunomodulation.

[43] Yang R., Zhang M.Q., Xue Y., Yang R., Tang M.M. (2019). Dietary of n-3 polyunsaturated fatty acids influence neurotransmitter systems of rats exposed to unpredictable chronic mild stress. Behav. Brain Res..

[44] Levin-Arama M., Abraham L., Waner T., Harmelin A., Steinberg D.M., Lahav T., Harlev M. (2016). Subcutaneous Compared with Intraperitoneal KetamineXylazine for Anesthesia of Mice. J. Am. Assoc. Lab. Anim. Sci..

[45] Du X.D., Yin M., Yuan L. (2020). Reduction of depression-like behavior in rat model induced by ShRNA targeting norepinephrine transporter in locus coeruleus. Transl. Psychiatry.

[46] Scheinert R.B., Haeri M.H., Lehmann M.L., Herkenham M. (2016). Therapeutic effects of stress-programmed lymphocytes transferred to chronically stressed mice. Prog. Neuropsychopharmacol. Biol. Psychiatry.

[47] Peng Z., Zhang C., Yan L., Zhang Y., Yang Z., Wang J., Song C. (2020). EPA is More Effective than DHA to Improve Depression-Like Behavior, Glia Cell Dysfunction and Hippcampal Apoptosis Signaling in a Chronic Stress-Induced Rat Model of Depression. Int. J. Mol. Sci..

[48] Zhang C., Zhang Y.P., Li Y.Y., Liu B.P., Wang H.Y., Li K.W., Zhao S., Song C. (2019). Minocycline ameliorates depressive behaviors and neuro-immune dysfunction induced by chronic unpredictable mild stress in the rat. Behav. Brain Res..

